# *PTCH1* is a reliable marker for predicting imatinib response in chronic myeloid leukemia patients in chronic phase

**DOI:** 10.1371/journal.pone.0181366

**Published:** 2017-07-13

**Authors:** Juan M. Alonso-Dominguez, Luis Felipe Casado, Eduardo Anguita, Maria Teresa Gomez-Casares, Ismael Buño, Francisca Ferrer-Marín, Alicia Arenas, Rafael Del Orbe, Rosa Ayala, Pilar Llamas, Rocio N. Salgado, Santiago Osorio, Pedro Sanchez-Godoy, Carmen Burgaleta, Ignacio Mahíllo-Fernández, Valentin Garcia-Gutierrez, Juan Luis Steegmann, Joaquín Martinez-Lopez

**Affiliations:** 1 Hospital Universitario Fundación Jiménez Díaz, Instituto de Investigación Sanitaria Fundación Jiménez Díaz (IIS-FJD), UAM, Madrid, Spain; 2 Hospital Virgen de la Salud, Toledo, Spain; 3 Hospital Universitario Clínico San Carlos, Madrid, Spain; 4 Hospital Universitario Doctor Negrín, Las Palmas, Spain; 5 Hospital General Universitario Gregorio Marañon. Instituto de Investigación Sanitaria Gregorio Marañón (IiSGM), Madrid, Spain; 6 Hospital Universitario Morales Meseguer,UCAM, CIBERER, Murcia, Spain; 7 Fundación Investigación Biomédica Hospital Universitario 12 de Octubre, Madrid, Spain; 8 Biocruces Health Research Institute,Barakaldo (Bilbao), Spain; 9 Hospital Universitario 12 de Octubre, Madrid, Spain; 10 Hospital Universitario Severo Ochoa, Leganés (Madrid), Spain; 11 Hospital Universitario Príncipe de Asturias, Alcalá de Henares (Madrid), Spain; 12 Hospital Universitario Ramón y Cajal, Madrid, Spain; 13 Hospital Universitario de la Princesa, Madrid, Spain; Universita degli Studi di Firenze, ITALY

## Abstract

Patched homolog 1 gene (*PTCH1*) expression and the ratio of *PTCH1* to *Smoothened* (*SMO*) expression have been proposed as prognostic markers of the response of chronic myeloid leukemia (CML) patients to imatinib. We compared these measurements in a realistic cohort of 101 patients with CML in chronic phase (CP) using a simplified qPCR method, and confirmed the prognostic power of each in a competing risk analysis. Gene expression levels were measured in peripheral blood samples at diagnosis. The *PTCH1*/*SMO* ratio did not improve *PTCH1* prognostic power (area under the receiver operating characteristic curve 0.71 vs. 0.72). In order to reduce the number of genes to be analyzed, *PTCH1* was the selected measurement. High and low *PTCH1* expression groups had significantly different cumulative incidences of imatinib failure (IF), which was defined as discontinuation of imatinib due to lack of efficacy (5% vs. 25% at 4 years, *P* = 0.013), probabilities of achieving a major molecular response (81% vs. 53% at first year, *P* = 0.02), and proportions of early molecular failure (14% vs. 43%, *P* = 0.015). Every progression to an advanced phase (n = 3) and CML-related death (n = 2) occurred in the low *PTCH1* group (*P*<0.001 for both comparisons). *PTCH1* was an independent prognostic factor for the prediction of IF. We also validated previously published thresholds for *PTCH1* expression. Therefore, we confirmed that *PTCH1* expression can predict the imatinib response in CML patients in CP by applying a more rigorous statistical analysis. Thus, *PTCH1* expression is a promising molecular marker for predicting the imatinib response in CML patients in CP.

## Introduction

At the beginning of this century, aberrant tyrosine kinase activity of the BCR-ABL1 oncogene protein was identified as a putative therapeutic target. This finding led to the development of imatinib, which revolutionized the treatment of chronic myeloid leukaemia (CML). However, roughly 25% of patients discontinue imatinib due to lack of efficacy [1]. A few years ago, two second-generation tyrosine kinase inhibitors (TKIs), dasatinib and nilotinib, were approved for first-line treatment, increasing the armamentarium available for patients diagnosed with CML in chronic phase (CP-CML) [2,3].

Currently, the best way to adjust treatment to fit the severity of the disease is by assessing the response at different milestones. One milestone is the *BCR-ABL1/ABL1* transcript level at 3 months; a level >10% 3 months after starting treatment has been related to a worse subsequent imatinib response [4]. However, whether a change in therapy is warranted based on this single determination is controversial [5]. Rather than switching the TKI early to reduce the *BCR-ABL1* burden rapidly, a better option could be selecting the appropriate TKI before starting treatment. Three different clinical scores (Sokal, EUTOS, and Hasford) are currently used to predict the outcome of patients diagnosed with CP-CML [6–8]. Although developed in the pre-TKI era, the Sokal and Hasford scores have retained their predictive power in patients treated with TKIs. The European Leukemia Net (ELN) states that a high risk classification in any of the scores should be considered a warning, but no clear recommendation has been made regarding treatment [5].

Several studies have defined molecular markers, such as *OCT-1* or *ABCB1* expression levels or polymorphic variants, that can predict the imatinib response in CML patients [9–11]. A molecular signature has also been sought with the same purpose [12–15]. Nevertheless, neither a single nor composite gene expression pattern has been generally adopted to guide first-line CML treatment. A previous study made comparisons among genes that overlap in expression profiles and genes highlighted as important in CML physiopathology or imatinib response prediction. Only *Patched homolog 1* (*PTCH1*) had a significant impact on predicting the response to imatinib [16]. PTCH1 is a component of the Hedgehog (Hh) signaling pathway, which is a key regulator of cell proliferation, cell surveillance, embryonic development, adult tissue homeostasis, and stem cell quiescence [17,18]. Different alterations in Hh have been described in basocellular carcinoma, medulloblastoma, rhabdomyosarcoma, lung cancer, gastrointestinal tract cancers, pancreatic cancer, lymphoma, and myeloma [19–24]. Smoothened protein (SMO), another component of the Hh pathway, is physiologically repressed by PTCH1. SMO has been identified as a molecular target, and different SMO inhibitors are being tested for the treatment of CML and other hematological and solid tumors [25,26].

In a subsequent study, the prognostic power of *PTCH1* was confirmed, but the calculated ratio of *PTCH1* expression to *SMO* expression had greater predictive potency [27]. The only cohort in these studies comprising off-trial patients and analyzed by conventional qPCR included 37 patients [16,27]. CML-related deaths and the rate of progression were clearly greater in that cohort than previously reported in CML [1]. Kaplan-Meier analysis was applied to analyze the different variables and three control genes (*GUSB*, *B2M*, and *18S*) utilized in the expression analyses [16,27]. In order to evaluate a prognostic test, it is important to test its performance in a cohort as similar as possible to the real scenario [28]. In addition, because CML is a chronic disease, competing risk has been accepted as a better option for analysis [29]. Finally, employing more control genes may produce a more consistent result, but that strategy is quite time-consuming, which restricts its applicability [30].

The present study aimed to confirm the feasibility of using *PTCH1* expression levels or the ratio of *PTCH1/SMO* expression as predictive CML markers in the clinical setting. We compared the prognostic power of the proposed predictors (*PTCH1* vs. *PTCH1/SMO*) in a realistic cohort using a simplified qPCR strategy, and an appropriate statistical analysis.

## Materials and methods

### Patients

**Fig 1:**
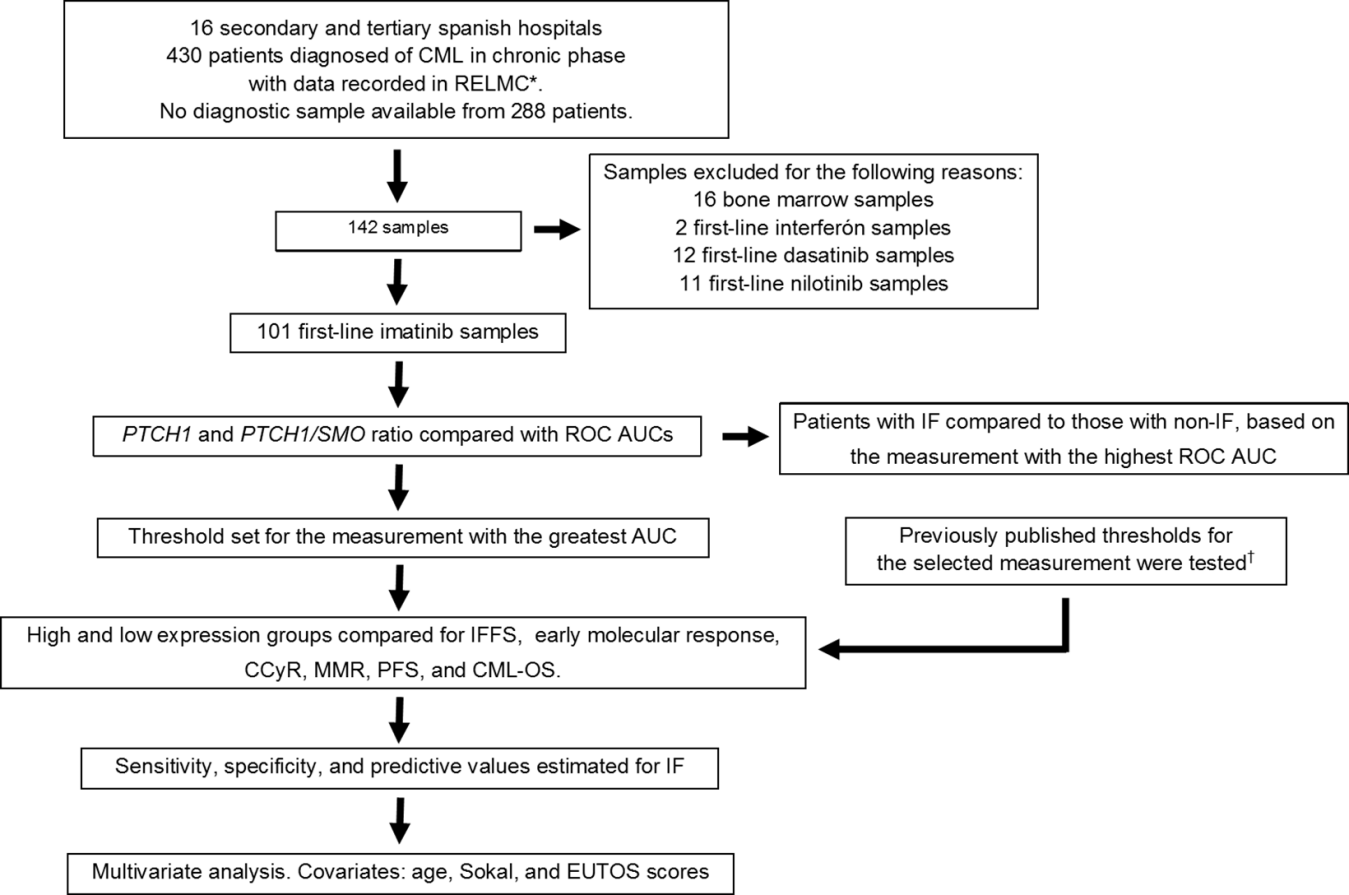
Flow diagram of sample selection and study design. RELMC, Spanish CML registry; IF, imatinib failure; IFFS, imatinib failure-free survival; CML-OS, overall survival related to CML. *Clinical data for 40 patients from two hospitals were collected *ad hoc*. †Groups established with previous thresholds were only compared in a univariate analysis.

This study included 101 patients diagnosed with CP-CML, as defined by ELN criteria, between 2002 and 2014. The patients were treated in 16 secondary and tertiary Spanish centers following international guidelines [5,31,32]. The patients were selected according to the availability of adequate sample material and follow-up data. A flow diagram of the sample selection and study design is shown in Fig 1. Clinical data were retrieved from the Spanish CML registry (RELMC) or collected *ad hoc* in two hospitals. Patients enrolled in a clinical trial were not included. All patients provided written informed consent for the use of their samples and clinical data for research purposes in accordance with the Declaration of Helsinki. The study was approved by the ethics committee of Hospital Doce de Octubre.

Patients were initially treated with 400 mg imatinib, and the dose was adjusted or the treatment discontinued depending on tolerance and the response following ELN recommendations. Patients who had received interferon or other pre-TKI treatments were excluded from the study. Bone marrow cytogenetics and molecular monitoring were carried out according to ELN recommendations [5,31–34].

### Samples and *BCR-ABL1* monitoring

All samples comprised peripheral blood anticoagulated with EDTA collected prior to imatinib treatment. Of the 101 samples collected, 50 were stored in the Hospital 12 de Octubre, Madrid, Spain, where molecular detection and monitoring of *BCR-ABL1/ABL1* was performed. For these samples, the RNA was extracted using Tri Reagent (MRC, Ohio, USA) and reverse transcription performed using the High Capacity cDNA Reverse Transcription Kit (Life Technologies, California, USA) according to the manufacturer´s instructions. *ABL1* served as a control gene, and the primers and probes were described elsewhere [35]. The laboratory employed an international scale (IS) conversion factor. The European Treatment and Outcome Study (EUTOS) recommendations were followed for molecular monitoring [36].

The remaining 51 samples underwent molecular monitoring in five different laboratories. Either RNA or cDNA extracted at the time of CML presentation was sent to the Hospital 12 de Octubre for enrollment in the study. RNA extraction was performed using Tri Reagent (MRC), the RNeasy Kit (Qiagen, Hilden, Germany), or the 16 LEV Simply RNA Blood Kit (Maxwell, California, USA). Reverse transcription was performed using the QuantiTect Probe RT-PCR Kit (Qiagen), the Transcriptor First Strand cDNA Synthesis Kit (Roche, Basel, Switzerland), or Superscript III (Invitrogen, Massachusetts, USA). The primers and probes were previously described [35]. Different calibrants were employed: BCR-ABL pDNA Calibrant (Sigma Aldrich, Missouri, USA), Qiagen Ipsogen BCR-ABL1 Mbcr IS-MMR Kit (Qiagen), or the ARQ IS Calibrator Panel (Asuragen, Texas, USA). In two of the five laboratories where 36 patients were followed, the GeneXpert Platform (Cepheid, California, USA) was used for *BCR-ABL1* detection and monitoring [37].

### qPCR

TaqMan gene expression assays (Life Technologies) were performed to evaluate the expression of *GUSB* (Hs00939627_m1), *PTCH1* (Hs00181117_m1), and *SMO* (Hs01090242_m1). Individual gene expression levels were measured as the mean quantification cycle (Cq) of two technical replicates, with a manual threshold assignment in every run. The 2^-ΔCq^ method was applied to calculate the relative expression of each gene normalized to the expression of *GUSB*. Outliers (>1.5 Ct difference among replicates) were discarded and the analysis repeated until precise quantification was achieved [38].

### Univariate analysis

We followed the reporting guidelines for tumor marker prognostic studies [28]. The prognostic powers of *PTCH1*expression, *SMO* expression, and the *PTCH1/SMO* expression ratio were evaluated by receiver operating characteristic (ROC) analysis. ROC curves were plotted to predict imatinib failure (IF), and confidence intervals (CIs) were estimated by a non-parametric method. For the measurement with the greatest area under the curve (AUC), we selected an optimal cut-off value that considered both the sensitivity and specificity of the ROC analysis. The levels of the selected measurement were compared between patients characterized as IF or no-IF using a Mann-Whitney test.

The primary endpoint of the study was imatinib failure-free survival (IFFS). IF was defined as previously described, except that non-CML-related deaths and discontinuation of imatinib due to side effects were considered as competing risks [1,16]. Therefore, IF was defined as the discontinuation of imatinib due to lack of efficacy. Secondary endpoints were the probability of achieving CCyR; the probability of achieving a major molecular response (MMR); overall survival related to CML (CML OS); the probability of achieving a *BCR-ABL1/ABL1* transcript level above or below 10% after 3 months of treatment; and progression-free survival (PFS), defined as survival without evidence of progression to an accelerated phase (AP) or a blast crisis (BC) [39]. All of the variables were analyzed on an intention-to-treat basis.

We analyzed *BCR-ABL1/ABL1* levels at 3 months using the classical 10% cutoff for the whole cohort, but we also performed an independent analysis with a threshold of 1.5% for patients analyzed by GeneXpert [40]. We employed the cumulative incidence procedure and the Fine-Gray test to estimate and compare the probabilities of IFFS, PFS, and CML OS for the high and low gene expression groups [41]. Non-CML-related deaths and changes from imatinib due to toxicity were considered competing risks in the analysis of IFFS. Only non-CML-related deaths were considered competing risks for the analysis of PFS and CML OS. We performed a Kaplan-Meier analysis and log-rank test to analyze the probabilities of achieving CCyR and MMR, and a chi-square test to evaluate the 3-month measurement variable. The sensitivity, specificity, and predictive values of individual genes were assessed and two-sided CIs calculated by the Wilson method.

### Validation of previously published thresholds

We tested the applicability of *PTCH1* expression thresholds established in previous studies, namely 0.418 and 0.51 [16,27]. We applied these thresholds and determined new groups, then repeated the univariate analysis.

### Multivariable analysis

A multivariate analysis was performed for the prediction of IF. We employed a Fine-Gray regression analysis with a forward stepwise procedure. The regression analysis also included previously reported prognostic factors at diagnosis: age, Sokal score, and EUTOS score. We applied standard boundaries for variable entry (0.05) and removal (0.10). Proportional hazard assumptions were checked by studying each regressor’s interaction with time [42]. No estimation of missing values was performed. The statistical analyses were performed using the SPSS version 19.0 statistics package (IBM, Armonk, USA) and multivariate analysis using Stata version 13.0 (Stata Corp, Texas, USA).

## Results

The characteristics of the cohort are provided in Table 1. The median follow-up was 2.8 years (range 0.2–12.6 years). Fourteen patients discontinued imatinib due to lack of efficacy. Five presented with primary resistance (i.e., failed to achieve CCyR with imatinib), and nine presented with secondary resistance (i.e., positive response followed by a loss of response). Among patients with secondary resistance, five achieved CCyR and four achieved MMR on imatinib, but subsequently lost the response. Twelve patients discontinued imatinib due to side effects.

**Table 1:** Patient characteristics.

Clinical Characteristic	Total	High *PTCH1* exp.	Low *PTCH1* exp.
	n = 101	n = 51	n = 50
**Median age, years**	55	55	55
**Range**	19–88	33–87	19–88
**Median follow-up, years**	2.8	3	2.7
**Range**	0.2–12.6	0.2–12.4	0.2–12.6
**Sokal risk score, n (%)**			
**Low**	43 (43)	19(38)	24 (48)
**Intermediate**	47 (47)	25 (50)	22 (44)
**High**	10 (10)	6 (12)	4 (8)
**EUTOS score, n (%)**			
**Low**	96 (96)	47 (94)	49 (98)
**High**	4 (4)	3 (6)	1 (2)
**Response at 3 months, n (%)**			
**<10% BCRABL/ABL IS**	41 (71)	24 (86)	17 (57)
**≥10% BCRABL/ABL IS**	17 (29)	4 (14)	13(43)
**Response at 1 year, n (%)**			
**CCyR**	57 (92)	28 (93)	29 (91)
**No CCyR**	5 (8)	2 (7)	3 (9)
**Patients on imatinib at last follow-up**	73	41	32
**Imatinib failure**	14	2	12
**Imatinib discontinuation due to side effects**	27	10	17
**Patients in MMR or a deeper level of response**	62	31	31
**Evolution to AP/BC**	1/2	0/0	1/2
**AlloBMT**	0	0	0
**Deaths**	6	3	3
**Deaths related to CML**	2	0	2

Exp, expression; IS, international scale; CCyR, complete cytogenetic response; MMR, major molecular response; AP, accelerated phase; BC, blast crisis; BMT, bone marrow transplantation. Data are presented as number of patients unless otherwise noted.

### Predicting the imatinib response

The median relative expression levels of *PTCH1* and *SMO* were 0.026 (range 1.87 ×10^−3^ to 1.69) and 8.8 ×10^−3^ (range 6.52 ×10^−4^ to 0.75), respectively. The AUCs for *PTCH1* and *PTCH1*/*SMO* were 0.72 (95% CI 0.6–0.84) and 0.71 (95% CI 0.54–0.88), respectively. Given the lack of improvement in predictive power and the need for quantification of an additional gene (i.e., *SMO)*, we determined that measuring *PTCH1* was optimal for the present study. We set the cut-off for relative *PTCH1* expression at 2.6 ×10^−2^, which was very close to the median gene expression. Consequently, 50 and 51 patients were included in the low and high *PTCH1* expression groups, respectively.

**Fig 2:**
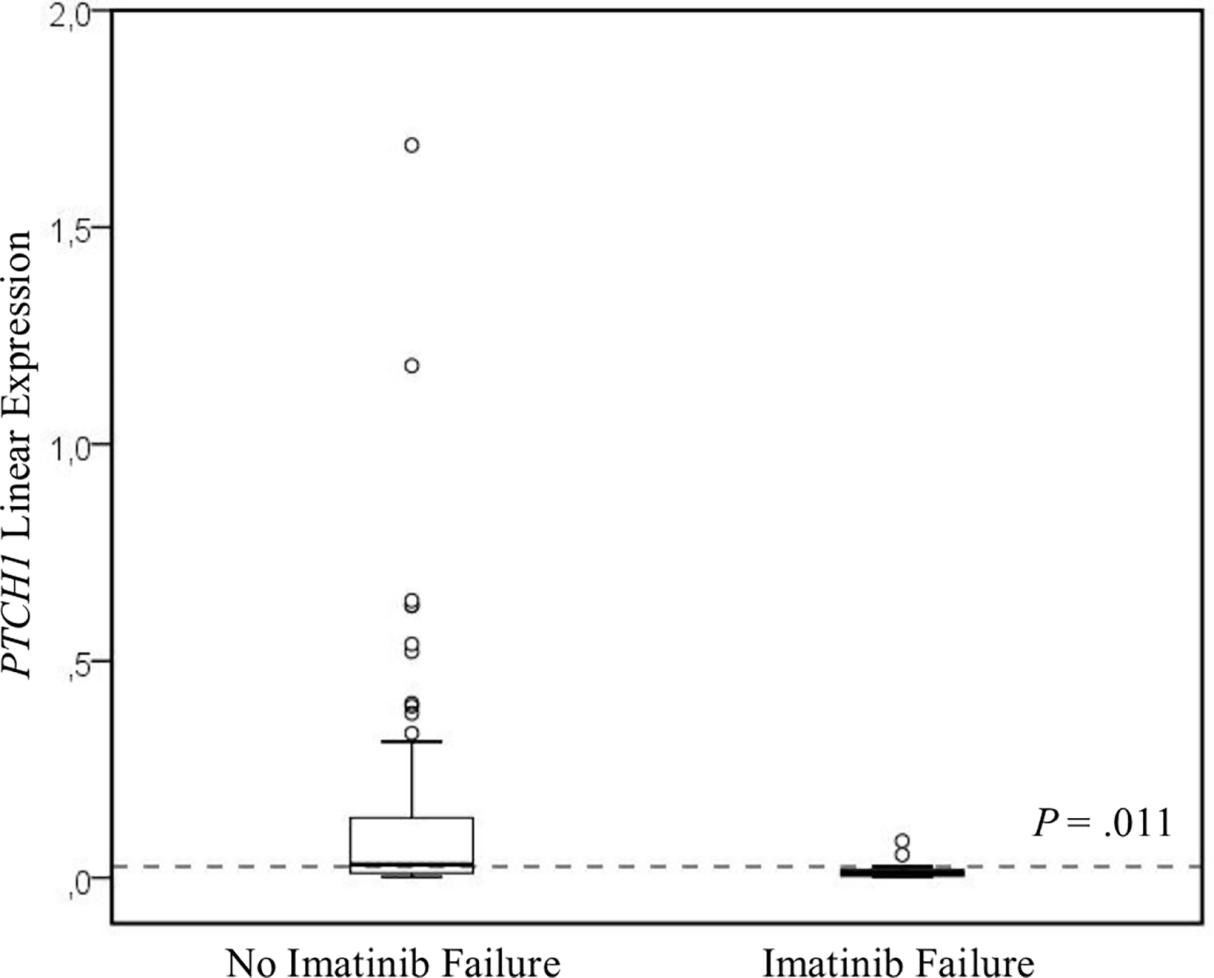
*PTCH1* expression in patients with and without imatinib failure (IF). The dashed line indicates the *PTCH1* expression threshold. IF group, n = 14; no-IF group, n = 87. Comparisons were performed with the Mann-Whitney test.

*PTCH1* expression is compared in patients with IF and those with no IF in Fig 2. Patients with IF had a median of *PTCH1* expression of 0.01, compared to 0.03 in the no-IF group (*P* = 0.011).

### Univariate analysis

**Fig 3:**
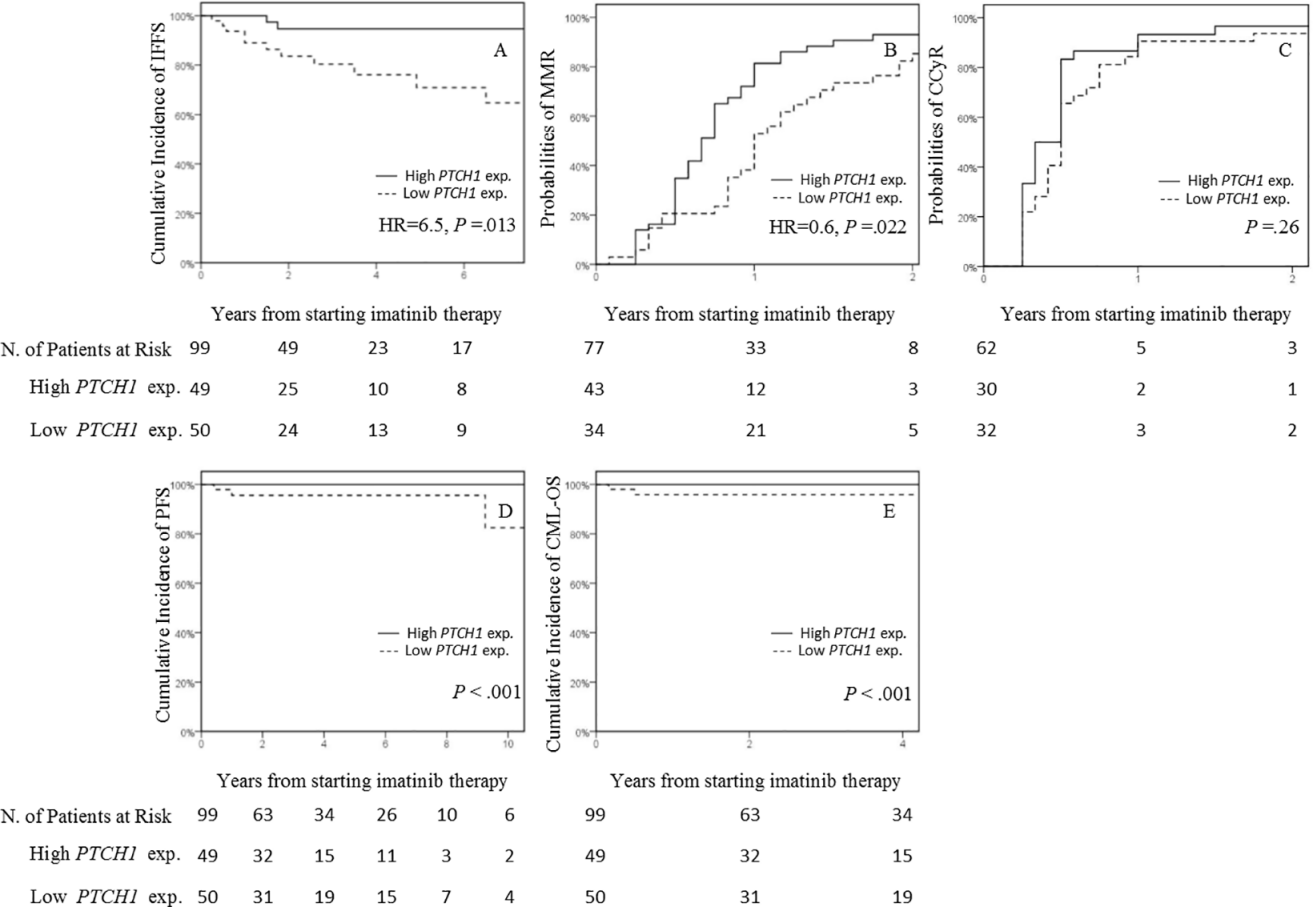
Cumulative incidence or Kaplan-Meier analysis of four different outcomes in the high and low *PTCH1* expression groups. HR, hazard ratio; exp, expression; IFFS, imatinib failure-free survival; MMR, major molecular response; CCyR, complete cytogenetic response; PFS, progression-free survival; CML-OS, CML-related overall survival; N, number. The solid line indicates high *PTCH1* expression (>0.0259) and the dashed line low *PTCH1* expression. Non-CML-related death was considered a competing risk in every variable analyzed by the cumulative incidence procedure. In addition, discontinuation of imatinib due to side effects was considered a competing risk for the analysis of IFFS. The HR is not shown if no event was recorded in any of the groups due to non-precise estimations. (A) IFFS analyzed by the cumulative incidence procedure and the Fine-Gray test. (B) MMR analyzed by Kaplan-Meier plot, log-rank test, and Cox regression (for HR estimation). (C) CCyR analyzed by Kaplan-Meier plot and log-rank test. (D) PFS analyzed by the cumulative incidence procedure and the Fine-Gray test. (E) CML-OS analyzed by the cumulative incidence procedure and the Fine-Gray test.

The cumulative incidence analysis and Fine-Gray comparison of IFFS, PFS, and CML-OS in the low and high *PTCH1* expression groups are shown in Fig 3. Two of the patients in the high *PTCH1* expression group and 12 in the low *PTCH1* expression group experienced IF. Patients in the high expression group who suffered IF achieved MMR and subsequently lost their response. Patients in the high *PTCH1* group had a 5% cumulative incidence of IF at 4 and 7 years, compared to a 25% and 36% cumulative incidence of IF in the low expression group at the same time points (*P* = 0.013, Fig 3A). Three patients progressed to AP or BC, and all three were in the low *PTCH1* expression group. The cumulative incidence of disease progression at 4 and 10 years was 0% in the high *PTCH1* expression group, compared to 4% and 18% in the low expression group (*P*<0.001, Fig 3D). Two of three patients who experienced disease progression died due to CML. The cumulative incidence of CML-related death was 4% and 0% at 4 years in the low and high *PTCH1* expression groups, respectively (*P*<0.001, Fig 3E).

Kaplan-Meier analysis and log-rank test were employed for the analysis of CCyR and MMR because no competing risks were recorded. MMR is compared between groups in Fig 3B. Seventy-seven patients had molecular monitoring records. The probability of achieving MMR at 1 year was 81% and 53% in the high and low *PTCH1* expression groups, respectively (*P* = 0.02). Cytogenetic data were available for 62 patients in the registry; 30 and 32 patients were in the high and low expression groups, respectively. No significant differences were found in the probability of reaching CCyR between groups (*P* = 0.26).

Fifty-eight patients, (28 in the high *PTCH1* expression group and 30 in the low *PTCH1* expression group, underwent 3-month *BCR-ABL/ABL1* monitoring. Based on the classical 10% *BCR-ABL/ABL1* cut-off, 4 (14.2%) and 13 (43.3%) patients in the high and low *PTCH1* expression groups, respectively, did not achieve *BCR*-*ABL1* ≤ 10% at 3 months (*P* = 0.015). We also observed significant differences when applying *BCR*-*ABL*/*ABL1* >1.5% (GeneXpert-corrected cut-off) or the conventional *BCR*-*ABL*/*ABL1* >10% cut-off, depending on the molecular technique applied for monitoring: 21% of the high expression group and 53% of the low expression group showed a non-optimal molecular response (*P* = 0.012) [40].

*PTCH1* measurement had a sensitivity of 85.7% (95% CI 60–96%), specificity of 56.3% (95% CI 46–66%), positive predictive value (PPV) of 24% (95% CI 14–37%), and negative predictive value (NPV) of 96.1% (95% CI 87–99%) for the prediction of IF.

### Validation of previously published thresholds

**Fig 4:**
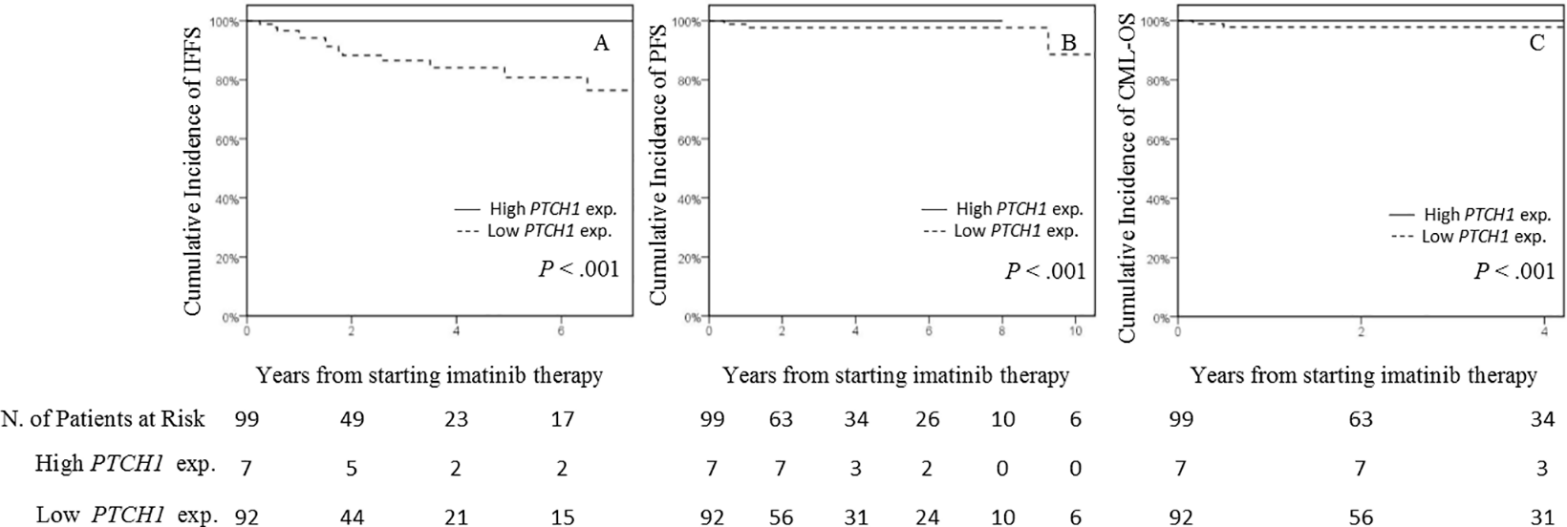
Cumulative incidence analysis of three different outcomes among high and low *PTCH1* expression groups established using previously published thresholds. HR, hazard ratio; exp, expression; IFFS, imatinib failure-free survival; MMR, major molecular response; PFS, progression-free survival; CML-OS, CML-related overall survival; N, number. The solid line indicates high *PTCH1* expression and the dashed line low *PTCH1* expression. Non-CML-related death was considered a competing risk in every variable analyzed by the cumulative incidence procedure. In addition, discontinuation of imatinib due to side effects was considered a competing risk for the analysis of IFFS. The HR is not shown if no event was recorded in any of the groups due to non-precise estimations. (A) IFFS analyzed by the cumulative incidence procedure and the Fine-Gray test. (B) PFS analyzed by the cumulative incidence procedure and the Fine-Gray test. (C) CML-OS analyzed by the cumulative incidence procedure and the Fine-Gray test.

When we applied the two different cut-offs previously published for *PTCH1* expression (0.418 and 0.51), we obtained the same number of patients in each group: 7 patients in the high *PTCH1* expression group and 94 in the low *PTCH1* expression group. IFFS, PFS, and CML-OS were significantly different between the two groups (Fig 4). No significant differences were found between the groups for CCyR (*P* = 0.6) or MMR (*P* = 0.8).

### Multivariable analysis

Ninety-eight patients were included in the multivariable analysis because three patients with missing values were excluded. Assumptions of the model were satisfactorily confirmed, and no transformation of any variable was required. *PTCH1* categorized expression showed a modest correlation (R = 0.269, P = 0.006) with age. Patients with high *PTCH1* expression had a mean age of 60 years vs. 52 in the low expression group. The *PTCH1* category of expression and EUTOS and Sokal scores were included in the final model (*P* = 0.008, HR = 7.7, 95% CI 1.7–33.3; *P* = 0.007, HR = 4, 95% CI 1.5–10.8; and *P* = 0.021, HR = 3.9, 95% CI 1.2–12.5, respectively). The *P-*value and HR shown for Sokal score were obtained from the comparison of low and high risk groups. No significant difference was found between the intermediate and low Sokal groups. In conclusion, the *PTCH1* category of expression was an independent prognostic factor for predicting IF.

## Discussion

This study included 101 patients diagnosed with CP-CML to obtain external validation and compare the predictive power of *PTCH1* expression and the *PTCH1/SMO* expression ratio. *PTCH1* expression had the greatest AUC for predicting IF, and the *PTCH1/SMO* ratio had a slightly reduced AUC. Given that we sought to reduce the number of genes to be quantified and the lack of improvement in the predictive power of the *PTCH1/SMO* expression ratio, we determined that measuring *PTCH1* was optimal for the present study [16,27].

Patients were classified based on a threshold of 0.0259 for relative *PTCH1* expression. High (n = 51) and low *PTCH1* expression (n = 50) groups had significant differences in the cumulative incidence of IFFS. The probability of attaining MMR was also significantly different between the two groups. We found that a greater proportion of patients in the low *PTCH1* expression group presented a non-optimal molecular response at 3 months. This finding was consistent when a cut-off of 10% of *BCR-ABL1/ABL1* was applied to the whole cohort or a combination of cut-offs was applied to subgroups (ELN-based qPCR and GeneXpert Platform) (37). Non-significant differences were found when CCyR probabilities were compared between the high and low *PTCH1* expression groups. A possible explanation for this finding was that the whole cohort had a rapid CCyR (median 6 months). In cases like this, a larger cohort needs to be analyzed to detect significant differences between groups.

Only three patients progressed to AP or BC, and two of these patients died from CML. All three patients who progressed and every CML-related death occurred in patients with low *PTCH1* expression. Similarly, in a previous report, every CML-related death occurred in patients classified in the low *PTCH1* expression group [16]. If no events occurred in one of the groups, a statistical estimation of HR was not precise, but as every patient who died from CML in both studies had low *PTCH1* expression, it does not seem to be a coincidence. Importantly, multivariate analysis showed that the level of *PTCH1* expression was an independent prognostic factor, separate from age, Sokal score, and EUTOS score, for predicting IF. Similar results were reported previously [16].

The ability of *PTCH1* expression to predict IF had a sensitivity, specificity, PPV, and NPV of 86%, 56%, 24%, and 96% respectively. Therefore, a patient with high *PTCH1* expression had a 96% probability of not discontinuing imatinib due to a lack of efficacy. Despite the high NPV, the performance of the test may not have been optimal given the low PPV result. When we consider that the EUTOS score, the only score developed in the imatinib era, had a sensitivity, specificity, PPV, and NPV of 15%, 95%, 31%, and 87%, respectively, for predicting a failure to achieve CCyR at 18 months in patients treated with 400 mg imatinib [8].

Taken together, our findings that the *PTCH1* measurement performed well in a largely different cohort extracted from secondary and tertiary hospitals with IF, progression, and CML-related death rates more similar to a real scenario adds external validity to previous results and suggests that the *PTCH1* measurement is a reliable prognostic test. However, like any gene expression study, a limitation of our study was the difficulty comparing our gene expression results to those of other laboratories. To address this issue, we validated previously published cut-off values for categorizing high and low *PTCH1* expression. These cut-offs were derived from qPCR analyses performed with a different set of primers and probes and normalization to three control genes instead of a single control gene [16,27]. When applied in our cohort, the two previously established cut-offs yielded groups with the same number of patients. Groups established with these previously published cut-offs, though very different in size, exhibited significant differences in IFFS, PFS, and CML-OS. By applying a previously published threshold, three patients who progressed to advanced phases and two patients who died from CML were also classified in the low *PTCH1* expression group. This finding is promising, but we are aware that reference materials should be developed, as done for *BCRABL/ABL1* quantification [43], in order to resolve standardization problems among laboratories and maximize the applicability of *PTCH1* measurement and, by extension, any other gene expression marker.

The Hh signaling pathway is a key regulator of stem cell quiescence, which increases with higher expression in the Hh pathway. PTCH1 acts by inhibiting SMO, which reduces activation of the GLI family of transcription factors, the effectors of the Hh pathway [18]. Although the CD34+ subfraction of stem cells does not uniquely represent the subpopulation of quiescent stem cells, no significant differences were found in *PTCH1* expression between CD34+ and CD34– populations derived from bone marrow CML samples [44]. Therefore, we hypothesize that the relationship found between an improved imatinib response and increased *PTCH1* expression in whole peripheral blood reflects a parallel increase in *PTCH1* expression in the subpopulation of quiescent leukemic stem cells. The augmented *PTCH1* expression would reduce Hh pathway activity, reducing the quiescence of CML stem cells; this effect would sensitize this cell population to imatinib treatment.

*PTCH1* expression does not seem to be related to the dasatinib response, and its relationship to the nilotinib response has not been tested [27]. If no relationship is found between *PTCH1* expression and nilotinib response, then *PTCH1* expression could guide first-line TKI treatment; if low *PTCH1*expression is detected at diagnosis, then a second-generation TKI would be a better treatment option.

In conclusion, we conducted a multicenter validation of previous *PTCH1* results with patients from a different country. Our cohort was more representative of the target population than previously studied cohorts. We implemented a simpler technique and a more rigorous statistical method than described previously [16,27]. Our results showed that *PTCH1* expression at diagnosis should be considered a promising molecular marker for predicting the probability of imatinib response in patients with CP-CML. These findings may facilitate clinicians’ ability to tailor a first-line TKI treatment to the individual patient.

## Supporting information

S1 File Excel file with clinical and gene expression data. (XLSX)
